# Memory Reactivation and Its Effect on Exercise Performance and Heart Rate

**DOI:** 10.3389/fspor.2020.00020

**Published:** 2020-05-29

**Authors:** Abhishek G. Dhawan

**Affiliations:** Agricultural Development Trust, Baramati, India

**Keywords:** neuronal ensemble, memory reactivation, THR, peak heart rate, exercise, aerobic

## Abstract

Neuronal ensemble and brain plasticity both play an important role in memory consolidation and subsequently memory reactivation. To date, many studies have been designed to study the effect of exercise, heart-rate variability, and other factors on brain plasticity and memory. Here, we present a case study in which we have demonstrated the effect of neuronal ensemble and memory formed during High-intensity aerobic training (VO2 max) and Target Heart-Rate (THR) training and the effect of reactivation of same memory on THR and performance. Of note is the fact that the reactivation and recreation of memory stimulus learned and formed during High-intensity training, such as place, time, odor, and other conditions, can elevate the THR to the same previous peak zone even at low intensity. This demonstrates that reactivation of previously acquired memory or using the stimulation from the neuronal ensemble of consolidated memory during the specific event of training may exert similar physiological effects on exercise or the body to those that are learned during the memory acquisition phase. Hence, as exercise has an effect on memory, the memories may have an effect on exercise performances.

## Introduction

Memories play an important role in cognitive as well as functional behavior. Exercise and memories have been an important part of studies in recent years. The effects of exercise on memory consolidation, Neurogenesis, and Neuroplasticity have been observed on a different scale. The paradoxical relationship has not been thoroughly observed or studied. The memory consolidation and reactivation incorporate neuro-cortical paradigms as well as physiological shifts. As motor and sensory inputs play an important role in memory formation, the studies on memory reactivation and its physiological effects on biological systems need to be understood as part of learning during memory encoding. To begin the case study, we have created an overview of the fundamentals of exercise and memory.

## Memory and Neuronal Ensemble

The memory is a collection of neurons and can be classified as declarative, procedural, or cognitive memory. The basic unit of memory in the brain is depicted as an Engram. An Engram is a unit of cognitive facts inside the brain, theorized to be representative of how memories are stored as biophysical or biochemical changes in response to sensory stimuli (Ungerleider et al., 2002; Herz et al., 2004; Moehring, 2019). The formation of memory is not just the acquisition of information, but it is more profoundly a three-step process: acquisition, consolidation, and reactivation. The neurons involved in memory formation are populated across the brain space, forming a neuronal ensemble. A neuronal ensemble (Qin et al., 1997; Sutherland and McNaughton, 2000) is a population of nervous system cells involved in a particular neural computation where computation could be a complete path of a different set of neurons involved in memory. Memory is often imbued with multisensory richness; the recall of an event can be endowed with the sights, sounds, and smells of its prior occurrence (Musso et al., 1999; Ramachandran and Rogers-Ramachandran, 2000; Flor et al., 2013). All types of sensory inputs are encoded in memory learned during acquisition. Memory Consolidation is the process of stabilizing a memory trace after the initial acquisition and making it available for long term archive and reactivation (Moorman and Miner, 1997; Lundborg, 2000; Banks, 2016). It is usually considered to consist of two specific processes: synaptic consolidation and systemic consolidation. It can be considered an interplay between multiple cortical brain regions, and it has a prominent role in the hippocampus.

The reactivation of memory triggers the whole path of an ensemble: neurons that wire together fire together. The retrieving of these memories involve the reactivation of neural ensembles that were established during learning (Kolb and Whishaw, 1998; Lane et al., 2009; Flor et al., 2013). The well set of an experiment performed using optogenetics demonstrates that reactivation of a set of neurons reactivates the same encoded emotional set (Laubach et al., 2000; Critchley et al., 2002; Sumbre et al., 2008; Smith et al., 2009). Any trigger to the partial sensory group may trigger the whole set of the ensemble, resulting in the complete retrieval of state or emotion.

When it comes to exercise, in this context, we have looked at aerobic exercise. *Aerobic exercise* mainly involves aerobic energy-generating process or exercise that involves primarily aerobic or free oxygen metabolic processes. This aerobic exercise could be categorized ranging from low intensity to high intensity. High-intensity exercises involve the peak performance of cardiorespiratory capacity. Peak Heart rate involves reaching above 85% of one's cardio capacity depending on age (Stiedl et al., 2004; Mahncke et al., 2006).

### Training Methods and Settings

The person involved in the case study was a 28-year-old male with a BMI of 20. The program was adapted for Target heart rate (THR) training by High-intensity Interval training-based treadmill running. The training aimed to achieve the THR in peak HR for the maximum time possible, thus improving VO2 max (Tayler et al., 2013; Thomas et al., 2016). The device used to track the Heart rate and Program data was a Fitbit Charge 2. The case study divided training into five phases, namely, the initial training phase, training phase, peak phase, weight training/detraining phase, and memory reactivation phase.

The maximum aerobic capacity or max heart rate (HR) was calculated by a formula created by Nes et al. (2013): HRmax = 211 – (0.64 × age), which is 193 bpm. The peak zone or maximum THR to peak zone (i.e., 85% and above of the maximum aerobic capacity) is >85% of 193. The training type used was High-Intensity Interval training (HIIT). High-intensity Interval training is built upon alternating between short, high-intensity bursts of energy with slower recovery phases throughout a single workout. Initially starting with steady-state cardio, this method gradually progresses to HIIT. It starts with an initial warm-up phase followed by alternating high-intensity running and slow walking recovery bursts; lastly, there is a cool-down phase. The aerobic detraining phase was achieved by inducing weight training. The weight training work out was low to mid-intensity weight training.

The main parameters considered for evolution were the heart rate zone and graph, the number of steps per exercise session, the calories burnt, and the duration of the exercise. The conditions set for training were considered to be the timing of training and the intensity of the treadmill, including speed in km/hr and inclination. The training was done in the morning session. Before training, the subject was regularly exercising/running for 20–30 min a day for 6 months. The average foot stride was of 2.5ft. The average room temperature varied from (22 ± 2) Celsius in winter to (31 ± 3) Celsius in summer. The activity tracker data report consists of the date, time, HR graph, Calorie burn graph, step count, HR zone, etc.

## Case Study

### Initial Training

**Figure 1:**
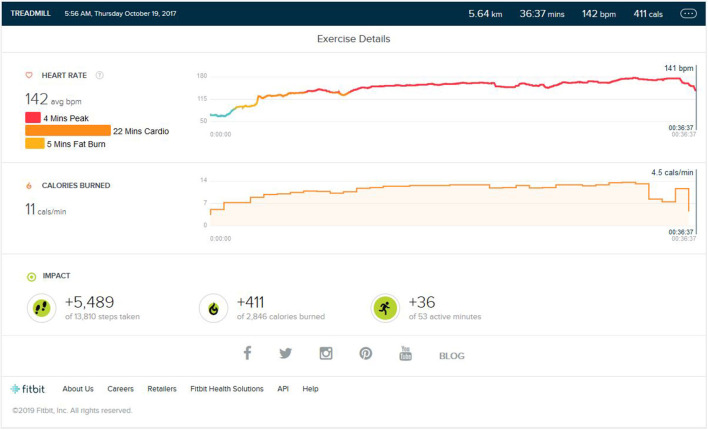
Analysis report during one of the initial training months.

This consisted of 5 km running on a treadmill within approximate 40 (±5) min. The initial warm-up burst was 3 KM/Hr to 6 KM/Hr walk for 5–7 min. The maximum speed of highest intensity per burst was 12 KM/Hr with every burst of 1 min, and the lowest was 6 KM/Hr for a low-intensity bout with every burst of 1 min. Total High- and Low-intensity alternating bursts was on average 25–29 min. The last 2–3 min phase consisted of the cool down. As shown in Figure 1, the average step count was (5,200 ± 400) steps per exercise session, summating to an average of 5 km on the ground running. The average calorie burn was (400 ± 30) calories. The peak heart zone (85 to 100 percent of the maximum HR) maintained was 4 (±2 min). The maximum maintained zone was the cardio zone (70 to 84 percent of your maximum heart rate). This training was varied to 4–5 days a week for nearly 7 months. The treadmill inclination varied by 2% (±1).

### Peak Phase

**Figure 2:**
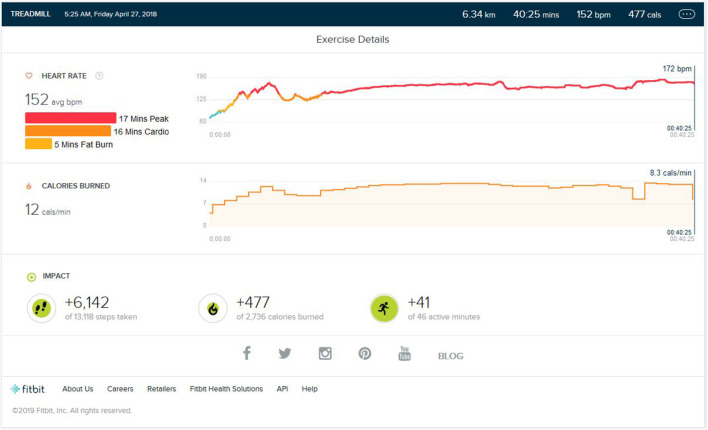
Analysis report during one of the Peak phase achieved months.

After the initial training, the peak performance stage was reached. As shown in Figure 2, the average step count was 5,600 (±500) steps per exercise session, resulting in an average of 5.5 (±0.5) km on the ground running. The average calorie burn was 470/±30 calories. The enhancement to VO2 Max was seen. The peak heart zone (85 to 100 percent of your maximum heart rate) that was maintained for 15 min (±4). The maximum maintained zone was the peak heart rate zone. The cardio zone was maintained for an average 15 (±4) min (70 to 84 percent of your maximum heart rate). All treadmill training parameters were, on average, the same as the initial phase.

### Post Peak Phase

**Figure 3:**
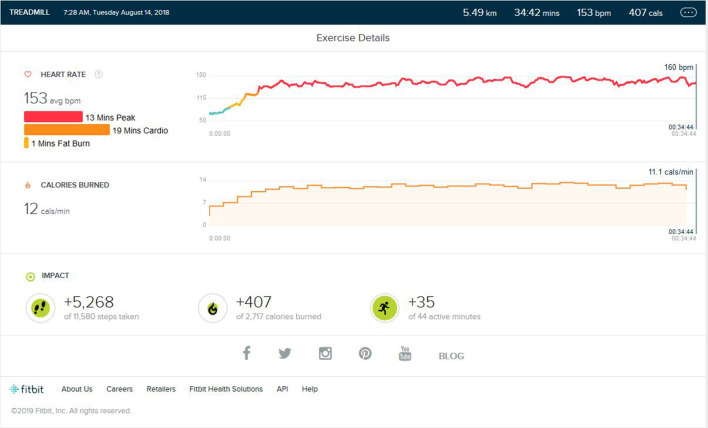
Analysis report during on one of the days of the maintenance phase months after months of peak phase being achieved.

After the peak performance stage was reached, the subject maintained a regular training schedule for 3–4 days a week, with a reduction in treadmill training parameters concerning the intensity of bursts, number of bursts per exercise, and high burst time duration. The main aim of the schedule was to maintain the peak heart performance achieved during training. As shown in Figure 3, the maintained VO2 Max was seen. The peak heart zone (85–100% of your maximum heart rate) maintained was 14 min (±4). The maximum maintained zone was the peak heart rate zone. The cardio zone was maintained for an average of 16 (±4) min (70 to 84% of your maximum heart rate).

### Weight Training Phase/Aerobic Detraining

**Figure 4:**
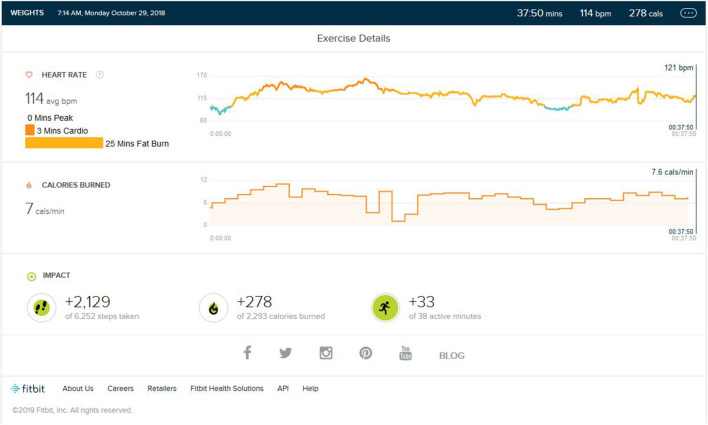
Analysis report on one of the days during weight training phase.

Following continuous induction of high-intensity aerobic exercise for almost 1.5 years, remarkable weight loss (10 ± 0.5 Kg) and subsequent muscle loss was seen. So, the partial detraining from aerobic exercise and added weight training was achieved. Weight training consisted of 2 days of weight training followed by cardio training for 1 day and a following 2 days of weight training. Weight training was low to mid intensity supplemented by a protein diet with 1–1.2 gm protein per kg in the natural form. No as shown in Figure 4, peak HR zone was maintained. The maximum HR zone maintained was the fat burn zone for an average of 22 (±10) min. Average exercise lasts for 40 min (±5). The average step count was 2,100 (±500) steps per exercise bout, coming to an average of 1.5 (±0.5) km. The average calorie burned was 250/±30 calories.

### Memory Reactivation

**Figure 5:**
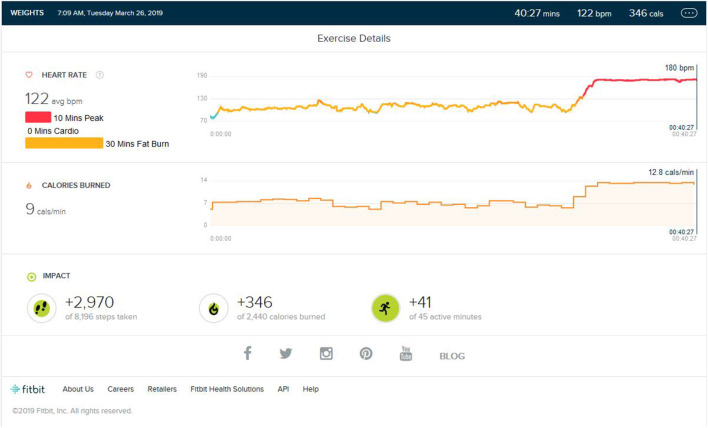
Analysis report on the addition of the same environment stimulus of treadmill jogging to weight training and HR raising to peak phase.

This was very important phase to be noted. After 3 months of treadmill activity of regular steady sate jogging/walking was added at a speed of 6 km/Hr for just 10 min (±3) at the end of weight training without any HIIT or high intensity burst, the heart rate suddenly peaked to the peak HR zone. The place and the time spent on the treadmill were all similar stimuli to that used during peak HR training. There was no high inclination of increment in the speed to reach peak HR. As shown in Figure 5, even the average distance covered after 10 min on the treadmill was not more than the 1.2 km average, and that is despite the total steps taken being close to 3,000 in the whole session.

To cross verify whether this was the effect of memory reactivation, i.e., the same stimulus as time, odor, temperature, place, and equipment, four different settings were tested. First, the treadmill session was added to the first part of weight training. Second, we tested the effect of running the whole session outdoors or in a completely different environment. Third, we changed the treadmill environment by changing the complete stimulus other than that previously used. Last, we carried out reverification after a few months under the same stimulus.

In the first trial, the result was the same as with the stimulus that was used during training when added at the start of weight training instead of at the end; HR was easily raised to the peak zone. It was tested for an average of 15 min. The heart rate was triggered to reach toward the peak HR zone in just couple of minutes. During the weight training session, it varied in the fat burn zone.

**Figure 6:**
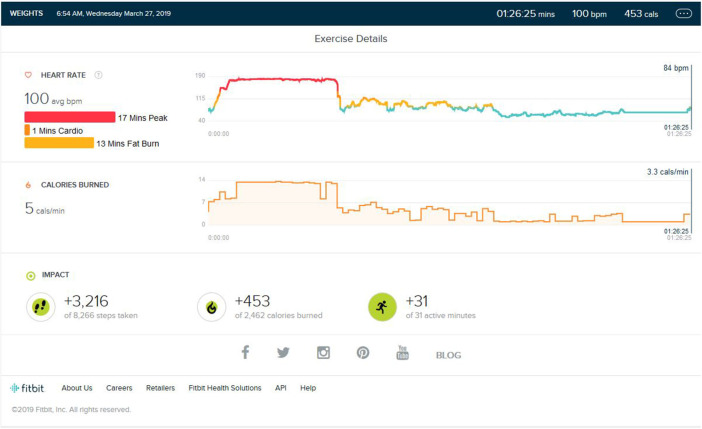
Analysis report on the addition of same environment stimulus of treadmill jogging at the start of weight training instead of the end of weight training and HR raising to peak phase.

As shown in Figure 6, during the second verification trial, the subject was made to run for 38 min (±2) in an outdoor non-similar stimulus environment with HIIT-type running. Of note was the fact that the heart rate remained in the cardio and fat burn zone and did not reach the peak HR. The distance was 4.6 km, and the step count was 4,870. Looking at distance covered, the step count, and the calorie burn graph, we can see that it was similar to the training or peak phase data, but changes in the environment resulted in different HR zones, and this was after nearly 5 months of aerobic partial detraining.

**Figure 7:**
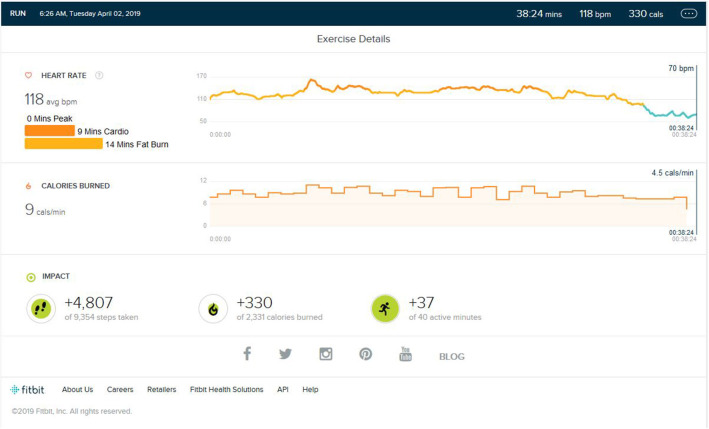
Analysis report on the addition of different environment stimulus in cardio training and HR not raising to the peak zone.

**Figure 8:**
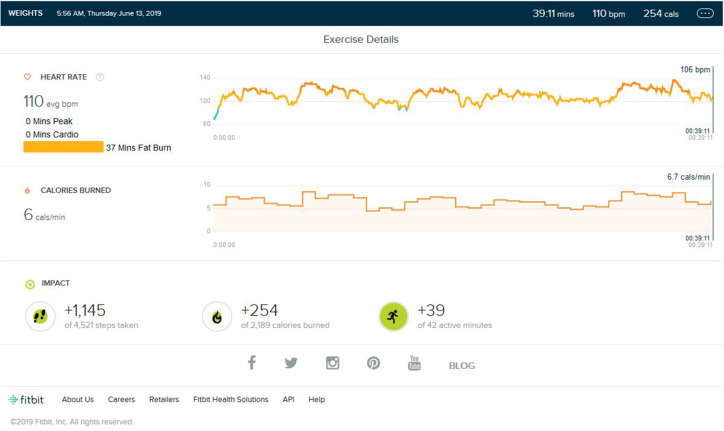
Analysis report on the addition of different environment stimuli to weight training and the HR not raising above the peak zone.

As shown in Figures 7, 8, during the third verification trial, yjr subject was made to run for 12 min (±2) in a non-similar treadmill stimulus environment with HIIT-type running during his weight training session of an average of 37 min. The location, equipment, and environment (Rasch and Born, 2007) was changed completely. Of note was the fact that the heart rate remained in the cardio and fat burn zone and did not reach the peak HR.

**Figure 9:**
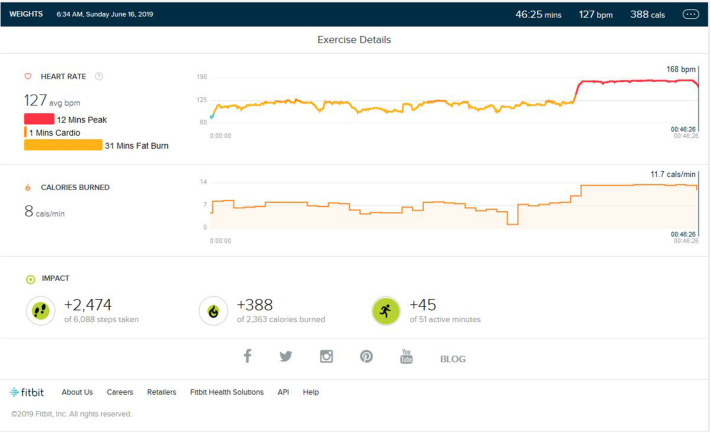
Analysis report reactivation of memory/environment stimulus during weight training and HR raising to peak zone, similarly to how it does during the memory acquisition phase.

In the fourth trial, the subject was weight trained in the same environment (Rasch and Born, 2007; Van Someren et al., 2011; Schedlbauer et al., 2015) after a few days of discontinuation with the addition of cardio training at the end of session, i.e., a similar treadmill stimulus environment with slow jogging of 6 km/Hr during the weight training session with the same place, time, equipment, etc, As shown in Figure 9, the heart rate was easily raised to the peak zone. It was tested for 12 min average. Heart rate was triggered to reach the peak HR zone in just a couple of minutes.

## Discussion

From this case study, it is of note that whenever the subject was in an environment (Rasch and Born, 2007; Wimmer and Büchel, 2015; Yanagisawa et al., 2016) that was similar to during training, the physiological response—in this case, the peak heart rate—was elevated to the same intensity zone, i.e., peak HR even with less or minimal physical effort, which was quite unexpected. The loss of efficiency after detraining was an expected factor that was observed in outdoor running after detaining, but the above result is opposite when it comes to peak HR during simple walking/jogging after weight training under the same stimulus. After partial detraining or weight training of about 6 months, as seen in second trial, the subject was not easily able to reach peak HR in a non-stimulus environment. The distinguishable thing is that, when the subject is confronted with same stimulus, even after same detraining phase, the subject was easily able to reach the peak HR. This is indicative of the predictable idea that the memory or neuronal reactivation may have physiological effects on the body that can extend to exercise performance. Many recent studies have linked the effect of memory reactivation to the physical responses (Hall et al., 1990; Flor et al., 2013; Banks, 2016) of body. Some studies, including that of V. S. Ramchandran et al., demonstrate that the pain acquired in disease and its memory during the pain phase have the same trigger effect, even after amputation of that part, e.g., phantom limbs (Gottfried et al., 2004; Flor, 2008) (the physiological effect of memory and pain). Brain plasticity is an important aspect of human physiological adaption (Rasch et al., 2007; Guan et al., 2016; Forte et al., 2019). Neural plasticity plays an important role in making the adaption of training into reflexes. Rigorous training for days results in improved cognitive reserve and elevated neuronal synaptic plasticity (Aglioti et al., 1994). Many recent studies have correlated the link between olfactory and other stimulus during memory consolidation to memory reactivation (Banks, 2016) by same stimulus (Armstrong and Maresh, 1998; Nyberg et al., 2000; Herz et al., 2004). The more intense the training, the stronger its memory formation. Fear or stress memories activate sympathetic responses in the body that include HR, dilation of the pupil, etc. (Cotman and Berchtold, 2002; Chamine and Oken, 2016; Corder et al., 2019), and, correlating all the above literature, it can be said that the memory reactivation could trigger the same physiological responses consolidated during acquisition. This demonstrates that reactivation of previously acquired memory or using stimulation of neuronal ensemble of consolidated memory during the specific event of training may exert similar physiological effects on exercise or the body that are learned during memory acquisition phase. Hence, as exercise has effect on memory, the memories may have an effect on exercise performances.

## Data Availability Statement

All datasets generated for this study are included in the article/supplementary material.

## Ethics Statement

The studies involving human participants were reviewed and approved by Agricultural Development Trust. The patients/participants provided their written informed consent to participate in this study.

## Author Contributions

The author confirms being the sole contributor of this work and has approved it for publication.

## Conflict of Interest

The author declares that the research was conducted in the absence of any commercial or financial relationships that could be construed as a potential conflict of interest.
